# Clinical lipidomics reveals high individuality and sex specificity of circulatory lipid signatures: a prospective healthy population study

**DOI:** 10.1016/j.jlr.2025.100780

**Published:** 2025-03-18

**Authors:** Jessica Medina, Nicolas Goss, Gonçalo dos Santos Correia, Rebecca Borreggine, Tony Teav, Zoltan Kutalik, Pedro Marques Vidal, Hector Gallart-Ayala, Julijana Ivanisevic

**Affiliations:** 1 Metabolomics and Lipidomics and Lipidomics Platform, Faculty of Biology and Medicine, University of Lausanne, Lausanne, Switzerland; 2 Institute of Reproductive and Developmental Biology, Department of Metabolism, Digestion and Reproduction, Imperial College London, London, UK; 3 March of Dimes Prematurity Research Centre at Imperial College London, London, UK; 4 Department of Computational Biology, Faculty of Biology and Medicine, University of Lausanne, Lausanne, Switzerland; 5 Department of Medicine, Internal Medicine, Lausanne University Hospital (CHUV) and University of Lausanne, Lausanne, Switzerland

**Keywords:** circulatory lipids, clinical lipidomics, HILIC-MS/MS, personalized signatures, prospective healthy population, sex differences

## Abstract

Abstract Lipid metabolism and circulatory lipid levels are tightly associated with the (cardio)metabolic health. Consequently, MS-based lipidomics has emerged as a powerful phenotyping tool in epidemiological, human population, and in clinical intervention studies. However, ensuring high-throughput and reproducible measurement of a wide panel of circulatory lipid species in large-scale studies poses a significant challenge. Here, we applied a recently developed quantitative LC-MS/MS lipidomics approach to a subset of 1,086 fasted plasma samples belonging to apparently healthy participants from prospective Lausanne population study. This high-coverage and high-throughput hydrophilic interaction liquid chromatography–based methodology allowed for the robust measurement of 782 circulatory lipid species spanning 22 lipid classes and six orders of magnitude-wide concentration range. This was achieved by combining semiautomated sample preparation using a stable isotope dilution approach and the alternate analysis of National Institute of Standards and Technology plasma reference material, as a quality control. Based on National Institute of Standards and Technology quality control analysis, median between-batch reproducibility was 8.5%, over the course of analysis of 13 independent batches comprising 1,086 samples collected from 364 individuals at three time points. Importantly, the biological variability, per lipid species, was significantly higher than the batch-to-batch analytical variability. Furthermore, the significantly lower between-subject (than within-subject) variability and unsupervised sample clustering demonstrated the high individuality and sex specificity of circulatory lipidome. The most prominent sex differences were reported for sphingomyelins and ether-linked phospholipids present in significantly higher concentrations in female plasma. The high individuality and sex specificity of circulatory lipidome constitute important pre-requisites for the application of lipidomics in next-generation metabolic health monitoring.

Lipids play a pivotal role in human metabolic health, serving as essential components of cell membranes, fuel sources or energy storage units, and mediators in cellular signaling in diverse metabolic processes (e.g., immune response, inflammation, amd insulin signaling). Consequently, lipidomics has emerged as a powerful phenotyping tool in biomedical research, as well as in human population and clinical intervention studies (1, 2, 3). Still, several major limitations remain to be addressed toward lipidomics utilization in clinical research and practice. First, the analytical platforms must allow for high-throughput and highly reproducible measurement of multiple sample cohorts, comprising thousands of samples, over extended periods of time (4). Second, data harmonization and cross-comparison between different studies and centers must be enabled through internal and external quality control (QC) strategies, using reference materials. This strategy is the outcome of international ring trials to ensure consistent and reliable reporting of quantitative data (5, 6, 7, 8). Finally, a critical amount of longitudinal data needs to be collected in apparently healthy populations, to evaluate biological variability of presumed biomarker analytes and establish reference values and intervals. This must be done while considering sex and age, as main determinants of circulatory lipid profiles (9, 10), prior to associating specific lipid species with clinical outcomes (e.g., cardiometabolic risk factors or disease incidence). These preliminary steps constitute the prerequisites for the evaluation of clinical utility of identified biomarkers which, themselves, should be deliverable in the form of clinical scores to facilitate interpretation and monitoring.

The main analytical platforms used for lipid analysis are direct infusion or direct injection analysis techniques, which include shotgun and differential mobility spectrometry with the Lipidyzer platform (11, 12), and hyphenated techniques, such as LC-MS. These analytical platforms enable accurate, reproducible, and quantitative lipid analysis (7, 13). However, the main advantage of LC-MS and other hyphenated techniques is reduced ion suppression, which improves sensitivity for the measurement of low-abundant species. Indeed, LC-MS is considered the most robust platform for lipid quantification, with the widest breadth of coverage. Lipid analysis is typically conducted using either reversed-phase liquid chromatography (RPLC) (3, 4, 14) or hydrophilic interaction liquid chromatography (HILIC) (15, 16, 17, 18). These differ mainly in the retention mechanism of lipids; using RPLC lipids are separated according to the acyl or alkyl chain length and number of unsaturations, whereas HILIC separates lipids by classes, through the interaction of the polar head group with the stationary phase. The retention mechanism of HILIC is advantageous for quantification due to coelution of endogenous lipid species and internal standards (IS), belonging to the same lipid subclass, and therefore, the ability to correct appropriately for the matrix effects. Therefore, direct injection analysis (e.g., using differential mobility spectrometry and multiple reaction monitoring–based Lipidyzer platform) and HILIC-based approaches are widely applied as the best compromise for high-throughput lipid quantification (7, 17, 19, 20), while RPLC-based methods are more fit for purpose of qualitative untargeted profiling for detailed and highly specific characterization of lipidomes (10, 14).

Recently, we developed and validated a method for quantifying more than 790 circulatory lipid species in 12 min run using an omics-scale (or high-coverage) HILIC-MS/MS approach (17). In this study, our aim was to demonstrate the robustness and reproducibility of the developed platform in a large-scale and longitudinal Lausanne population study (13 batches comprising 1,086 samples from 364 participants, https://www.colaus-psycolaus.ch) with apparently healthy participants at the baseline. In addition, we have evaluated the between- and within-individual variability of measured lipid species while considering the effect of sex and age as main determinants of circulatory lipid profiles.

## Materials and methods

### Chemicals, reagents, and IS mixture preparation

The chemicals and reagents and preparation of the IS mixture are described in the Supplementary material (supplemental Table S1).

### Semiautomated sample preparation

Dried ISTD mixture (75 species) was reconstituted by adding 25 *μl* of plasma sample and 100 *μl* of 2-propanol (IPA) for lipid extraction and protein precipitation using the Bravo system (Agilent Technologies, Santa Clara, California). The extracts were shaken (5 min at 1,000 rpm) and centrifuged (15 min at 20,000 *g* at 20°C) (Hermle Z 326 K, Gosheim, Germany). Finally, the supernatant (75 *μl*) was transferred, using the liquid handler, to a new 96-well plate (Thermo Fisher Scientific, San Jose, CA) for LC-MS/MS analysis. For more details on sample preparation, please refer to Medina *et al.* (17).

### LC-MS analysis

Plasma extracts were analyzed by HILIC-MS/MS, using a dual-column setup on a Vanquish™ Duo UHPLC System coupled to a TSQ Altis triple-stage quadrupole (Thermo Fisher Scientific, San Jose, CA) operating in positive and negative ionization modes. Chromatographic separation, as described previously (21), was carried out on an Acquity Premier BEH Amide column (1.7 μm, 100 mm × 2.1 mm I.D., Waters, Milford, MA). A dual-column setup allowed for the re-equilibration of the first column while analyzing the sample on a second column, enabling analysis of each sample in 12 min. Optimized lipid-dependent parameters were used for data acquisition in timed-selected reaction monitoring mode (transitions are provided in supplemental Tables S2 and S3).

### Initial qualitative screen followed by high-throughput quantification

The initial screen comprising 2,441 lipid species distributed across eight methods in positive and in negative ionization mode was performed on a pooled sample, prepared from 84 randomly selected participants (10 μl from the baseline and follow-ups), representative of Lausanne population. Nine hundred thirty-four lipids (supplemental Tables S2 and S3) with a coefficient of variation (CV) lower than 20% (across five replicate injections of pooled sample during the initial qualitative screen) were included to the targeted list of a 12-min quantification method as described above. Please see supplemental Table S4 for 96-well plate design, including blank extracts, samples and National Institute of Standards and Technology (NIST) 1950 reference material as external QC samples.

### Standard reference material (SRM 1950 plasma) as long-term external QC

**Fig. 1:**
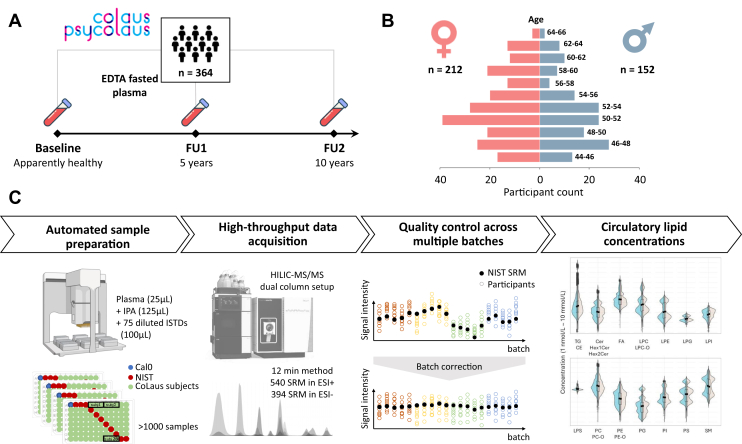
Lipidomic profiling in the context of large-scale population studies. A: Lipid profiling of 1,086 blood plasma samples collected from 364 participants of CoLaus cohort, over 10 years (two follow-ups). B: Apparently healthy population structure at baseline. C: Analytical workflow from automated sample preparation and high-throughput data acquisition to signal intensity drift correction, quality assessment, and concentration reporting.

Standard reference material for human plasma NIST SRM 1950 approved by the NIST (Gaithersburg, Maryland) was purchased from Sigma-Aldrich. NIST plasma was used as external QC and analyzed periodically, every 12 samples, within each sample batch (Fig. 1 and supplemental Table S4).

### CoLaus study

The study used data from the CoLaus|PsyCoLaus cohort (https://www.colaus-psycolaus.ch), a prospective study conducted in the population of Lausanne, Switzerland. A subset of apparently healthy participants followed over 5 and 10 years’ time (n = 1,086, 152 males aged 52.3 ± 5.2 and 212 females aged 53.22 ± 5.2 at the baseline) were selected for the investigation of intraindividual and interindividual variability and sex differences across plasma lipidome.

### Ethical statement

The institutional Ethics Committee of the University of Lausanne, which afterward became the Ethics Commission of Canton Vaud (www.cer-vd.ch) approved the baseline CoLaus study (reference 16/03). The approval was renewed for the first (reference 33/09) follow-up. The approval for the entire CoLaus|PsyCoLaus study was confirmed in 2021 (reference PB_2018–00038, 239/09). The full decisions of the CER-VD can be obtained from the authors upon request. The study was performed in agreement with the Helsinki declaration and its former amendments and in accordance with the applicable Swiss legislation. All participants gave their signed informed consent before entering the study.

### Block randomization

Samples were first randomized to have similar proportion of males and females in each batch. The samples belonging to same individual were acquired within the same batch, in randomized order (i.e., time points) (22).

### Data preprocessing

Raw data files were processed using Trace Finder Clinical Research 4.0 (Thermo Fisher Scientific). Peak area integration was manually curated. Lipid concentrations were estimated as the peak area ratio between the analyte and the most structurally similar IS multiplied by its known spiked concentration.

### Isotopic overlap correction

The contribution of heavy isotopes to the SRM transition intensities depends on their location in the molecule with respect to the fragmentation pattern. Correction for isotopic overlap based on lipid class separation in SRM acquisition mode was performed using the Shiny app LICAR (https://slinghub.shinyapps.io/LICAR/) developed by Gao *et al.* (23) at the Singapore Lipidomics Incubator (SLING).

### Batch correction

Signal intensity drift correction between different batches was performed using linear regression models predicting each lipid’s concentration as a function of batch (i.e., lipid ∼ batch). Comparison with models including terms for intrabatch and general run-order intensity drifts did not support the need for these extra terms. Each model was fitted only to the NIST QC samples, and then the estimated batch means were subtracted from the corresponding samples. Data was recentered by adding the grand mean of all batch concentration estimates for the NIST QC samples.

### Lipid nomenclature

We used the lipid nomenclature according to the latest Lipid Maps update by Liebisch *et al.* (24) Following abbreviations are used for each lipid class: CE = cholesteryl ester, DG = diacylglycerol, TG = triacylglycerol, Cer = ceramide, dhCer = dihydroceramide, HexCer = hexosylceramide, Hex2Cer = dihexosylceramide, SM, LPC = lyso phosphatidylcholine (LPC = acyl or ester-linked, LPC-O = alkyl or ether-linked), LPE = lysophosphatidylethanolamine, PC = phosphatidylcholine (PC = diacyl or ester-linked, PC-O = alkyl or ether-linked), PE = phosphatidylethanolamine (PE = diacyl or ester-linked, PE-O = alkyl or ether-linked), LPI = lysophosphatidylinositol, PI = phosphatidylinositol, LPG = lysophosphatidylglycerol, PG = phosphatidylglycerol, LPS = lysophosphatidylserine, PS = phosphatidylserine, FFA = free fatty acid, GP = glycerophospholipid, and SP = sphingolipid.

### Data analysis

Data tables obtained by the analysis of NIST plasma QCs and CoLaus plasma participants were analyzed with the R software version 4.3.2 (http://cran.r-project.org/). Linear mixed effect models were used to model sex differences in lipid concentration while adjusting for the effect of age and the longitudinal design. The dependent variable (i.e., lipid concentration) was standardized after log-transformation with addition of a small constant c = 1 and modelled as a function of sex and age with an interaction term between these covariates (fixed effects). Longitudinal intraparticipant trends were modeled using a random effect structure containing random slopes for age and random intercepts for each participant. Linear mixed effect models were fitted with lme4 using restricted maximum likelihood. Calculation of mean and trend estimates, contrast coding, and Welch’s *t*-tests were performed with the *emmeans* package (1.10.3), using the Kenward-Roger approximation for degree of freedom estimation. Obtained *P* values were corrected for false discovery rate with the Benjamini–Hochberg method (*q* value < 0.05). Intraclass correlation coefficients (ICCs) were estimated as the ratio of random intercept variance to the total variance (residual plus the random effect variance), using a mixed effect model with sex as a fixed effect and a random intercept per participant. Principal component analyses and hierarchical clustering were performed using *pcaMethods* package (1.90.0), on the mean-centered and UV-scaled data. The *ape* package was used to plot the results from hierarchical clustering. Plots were created using ggplot 2 (3.5.1).

## Results

### High-throughput clinical lipidomics platform for large-scale population phenotyping

We acquired circulatory lipid profiles of 1,086 plasma samples collected from 364 individuals (in fasted state) who participated in adult Lausanne population study over a 10-year period (Fig. 1A, B, baseline and two follow-ups over 5 and 10 years’ time). As displayed on Fig. 1C, the samples were prepared in a randomized and semiautomated manner, using a liquid handling platform, in 13 batches (or 96-well plates) together with blank extracts and NIST reference material as external QC. We applied the single-step extraction with IPA, previously validated as the best compromise in terms of extraction efficiency and reproducibility (21). For lipid measurement, the stable isotope dilution approach was applied using a mixture of 75 ISs spanning the entire panel of diverse lipid subclasses with multiple representatives for each class (see supplemental Table S1). Lipid separation and data acquisition was achieved using our validated, high-coverage (englobing 884 lipid species, supplemental Tables S2 and S3) HILIC-based targeted method in SRM mode with a total run time of 12 min (17). Data quality assessment, including measurement precision, was ensured using repeated injections (every 12 samples) of NIST plasma reference material within each and across all sample batches. An example of the 96-well plate design can be found in supplemental Table S4. Importantly, the instrument status (i.e., the signal intensity and overall function) was monitored using the above specified IS mixture as a system suitability test prior to sample preparation.

### QC and batch-to-batch reproducibility assessment

**Fig. 2:**
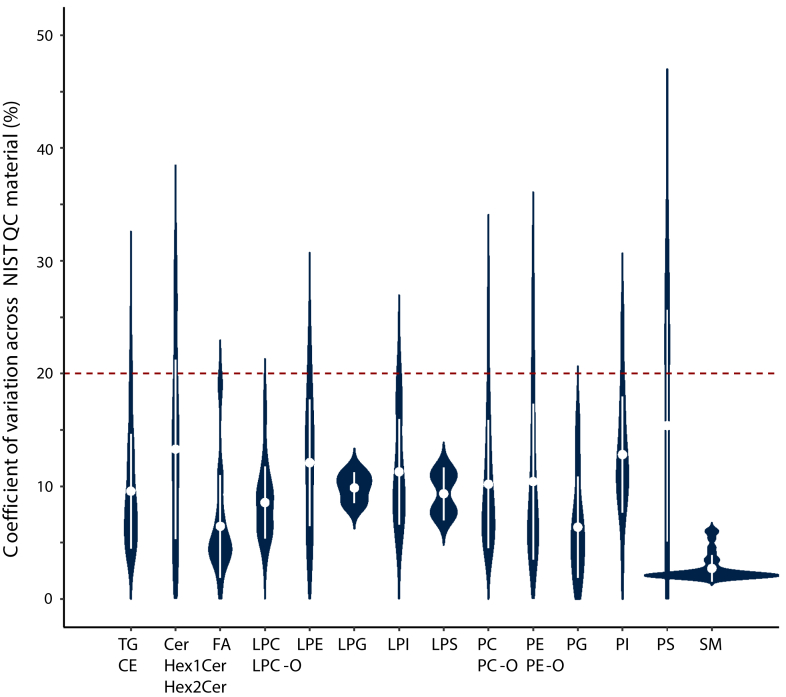
Analytical batch-to-batch variability across measured lipid classes. Measurement precision illustrated through the distribution of coefficients of variation per lipid subclass. Violin plots: white dot and line represent the mean and SD. Data table with values for each lipid species is provided in supplemental Table S5.

**Fig. 3:**
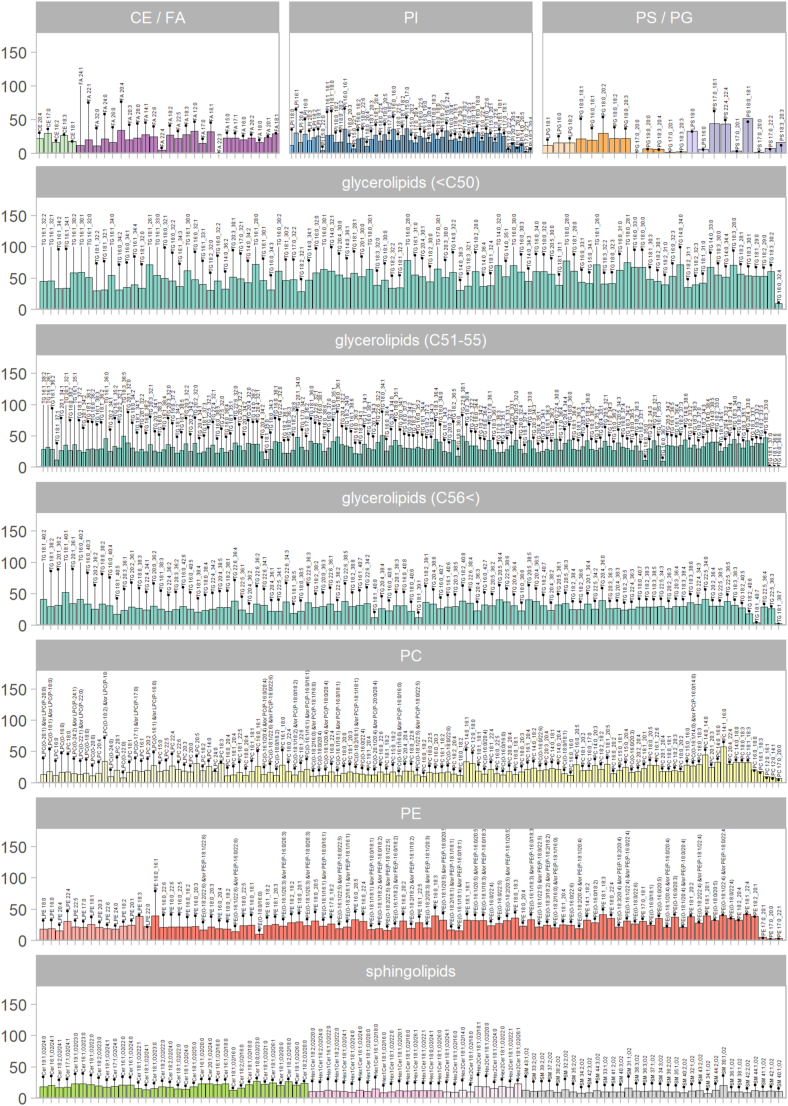
Biological variability per lipid class and species. The bar plots represent the intraindividual (or within-subject) and the lollipops, the interindividual (or between-subject) coefficients of variation (%). Data with values for each lipid species and lipid (sub)class are provided in supplemental Table S5. Lipid names are plotted above the lollipops.

Overall, the ISs allowed for the correction of matrix effects and data normalization with respect to variations in instrument sensitivity over time. However, signal intensity drift over time is inherent to MS. Owing to the periodical analysis of NIST reference material, we were able to correct, specifically for between-batch (or interbatch) drift (median CV = 8.5%) on a species-specific basis (supplemental Fig. S1). The drift correction was performed using linear regression modeling (see Materials and Methods) as the signal exhibited linear response over time (i.e., there was no need for the application of LOWESS for drift correction as usually done in untargeted assays). The between-batch signal intensity drift correction using NIST QCs allowed us to recover the additional 102 lipid species measured with good reproducibility (<30%). As a result, based on 143 NIST plasma QC samples over the course of analysis of 13 independent batches (1,086 samples), among 884 quantified lipids which passed the filter for isotope overlap correction, 782 were retained (based on interbatch precision) for further statistical analysis (Fig. 2, supplemental Table S5). The species which did not pass this QC filter were mainly among the coeluting neutral lipids, including CEs, DGs, and TGs. The exceptions were also PSs whose signal was at the limit of detection in NIST plasma. Nevertheless, their measurement was robust in the plasma samples of CoLaus participants, the reason why multiple PS and some other lipid species with CV up to 30% were kept for further statistical evaluation. Importantly, this analytical variability was evaluated to be, at least, two to three times lower than intraindividual and interindividual biological variation, depending on the lipid class and species. Moreover, for most species the intraindividual variation was also consistently lower than the interindividual variation (Fig. 3), suggesting that individual lipid profiles are distinct and stable over time.

### Circulatory lipid signatures show high individuality

**Fig. 4:**
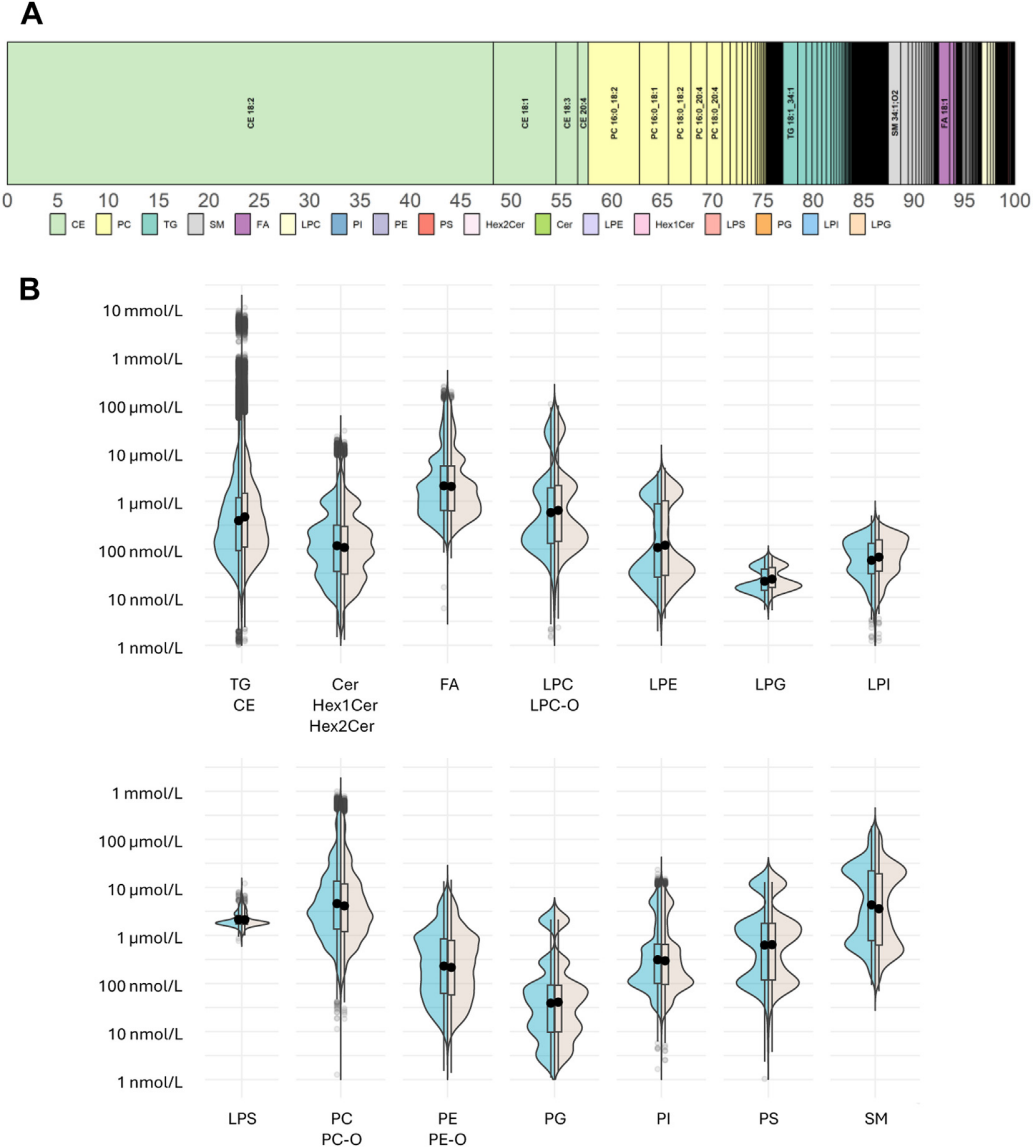
Relative abundance and estimated concentration range of measured lipid classes and species. A: Lipid signature showing the relative abundance of measured lipid species and classes. B: Sex-stratified concentration ranges of measured lipid classes. Box plots: Black dots represent the means for females (left) and males (right), boxes represent the range between the 25^th^ and 75^th^ percentile, and the whiskers represent the minimum and maximum values. The outlier values (>1.5 × interquartile range) are plotted as individual points. The mean concentrations of every measured lipid species are provided in supplemental Table S6.

Using the described analytical workflow, we were able to measure with high precision, in each individual’s sample, a wide panel of 782 lipid species, belonging to 22 lipid subclasses, and spanning six orders of magnitude-wide range of concentrations (Fig. 4A, B). The acquired signatures were characterized by 371 neutral lipids (including DG, TG, and CEs), 94 SPs (including Cer, dihydroceramides, HexCers, and SMs), 235 glycerophospholipids (including PGs, PCs, PEs, PSs, and phosphatidylinositols), in addition to 56 lysophospholipids (LP) (including lysophosphatidylglycerol, LPC, LPE, lysophosphatidylinositol, and lysophosphatidylserine), as well as 25 FFAs. The CEs were present in the highest concentrations (mmol levels) and the PGs and HexCers were measured in the lowest concentrations (low nanomolar levels, Fig. 4 and supplemental Table S6). As depicted in the circulatory lipid signature (illustrated as a “barcode” on Fig. 4A), the CE, PC, and TG represent the most abundant lipid classes accounting for 87% of total circulatory lipid content. SM, FFA, PE, LPC, and DG contribute around 12%, and the remaining lipid species account for less than 1% of circulatory lipid signature. Importantly, based on NIST SRM 1950 analysis, the measured concentration values are in accordance (i.e., in the same range) with previously reported analytical consensus values in interlaboratory trials carried out by Lipid Maps, Bowden *et al.*, Ghorasaini *et al.*, and recently Torta *et al.* focusing on Cer (supplemental Table S7, supplemental Fig. S2) (6, 7, 25, 26). For specific lipid classes, the reported concentrations might have been overestimated or underestimated (e.g., PE and LPEs in Lipid Maps study, TGs in Bowden *et al.*), this remains to be further evaluted in interlaboratory trials.

**Fig. 5:**
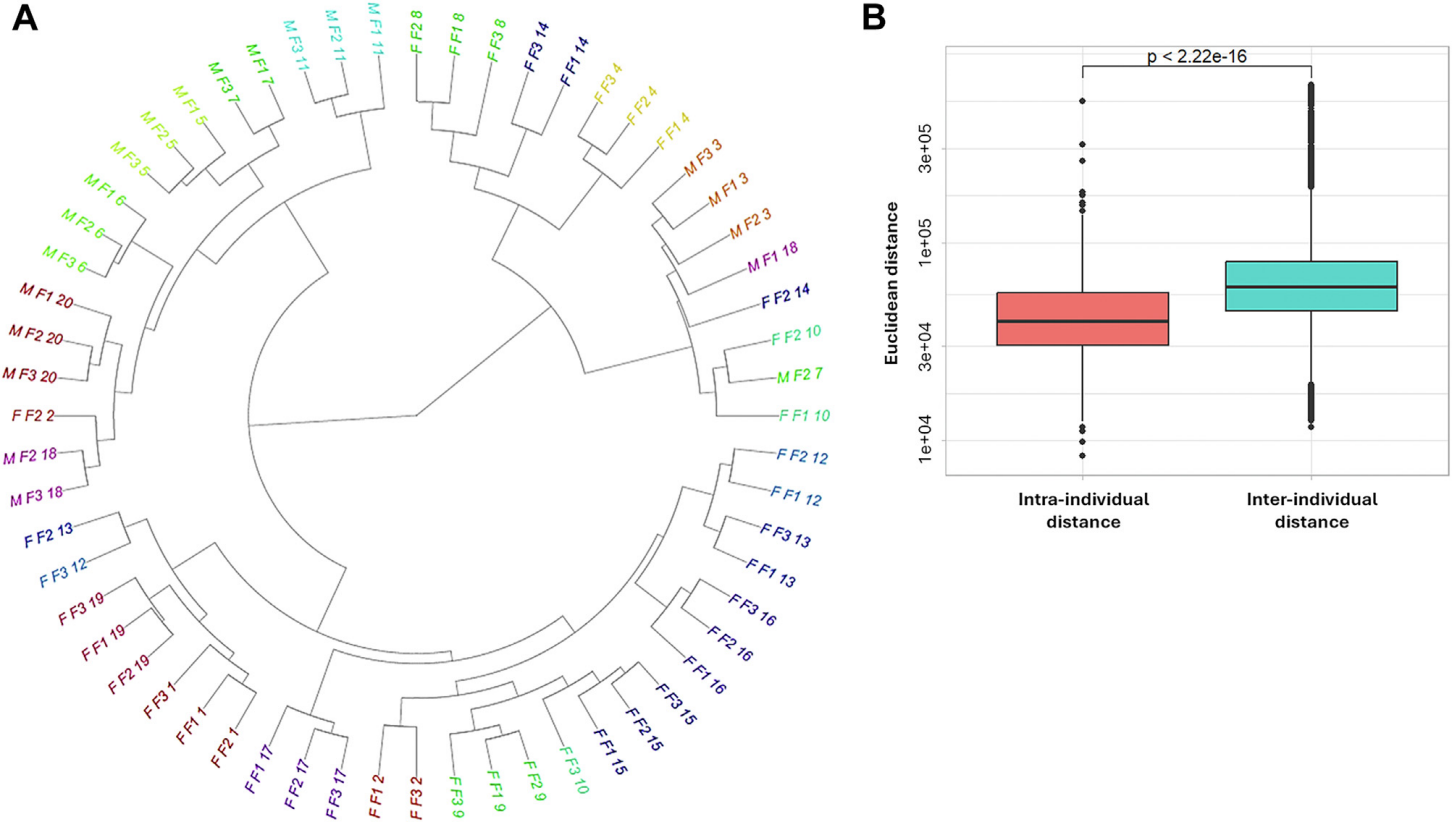
Individuality of lipid signatures. A: Hierarchical clustering showing the distances in lipid signatures from 20 randomly selected participants (60 samples from three time points, M-males and F-females) across the baseline (F1) and follow-ups (F2, F3). Samples from same individual are represented in same color tone. B: Boxplots report the average distance between triplets within-participant versus between-participant distance. The intraparticipant and interparticipant distances were compared using a two-sided *t*-test.

These comprehensive lipid signatures have shown a high level of individuality, with samples from same individual, collected over 10 years period, clustering together (with few exceptions). This is highlighted by unsupervised hierarchical clustering of sixty sample from randomly selected twenty participants (Fig. 5A). Similar results were obtained through successive resampling of different individuals. Accordingly, as shown in Fig. 3, the overall intraindividual distance was consistently and significantly lower than the interindividual distance (*t*-test, *P* < 2.22e-16, Fig. 5B). This longitudinal intraparticipant trend was further modeled using a random effect structure containing random slopes for age and random intercepts for each participant. The resulting ICC allowed us to evaluate the proportion of total variation explained by the participant in the cohort to assess stability over time and per lipid class (supplemental Table S8, supplemental Fig. S3). As shown in supplemental Fig. S3, for most lipid species (across multiple lipid classes), the ICC was >0.5. This strong intraindividual correlation points toward the high individuality with the individual, on its own (as a factor), explaining a high portion of variance in lipid signatures.

### Acquired lipid signatures are sex specific

**Fig. 6:**
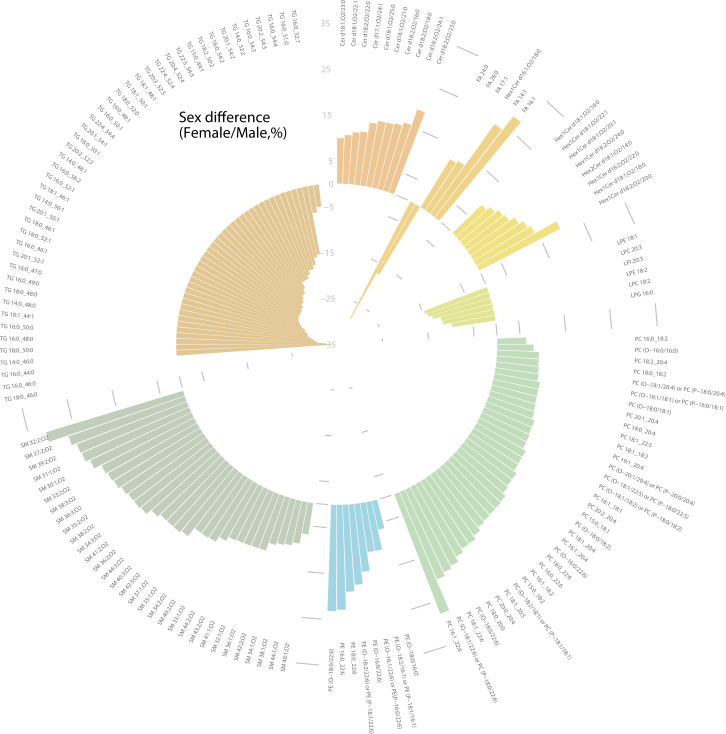
Sex differences across the human lipidome were revealed in a subgroup of clinically healthy participants from the CoLaus Study (www.colaus-psycolaus.ch/). The circular bar plot is organized by lipid class, displaying 147 lipid species that showed significant sex differences with an adjusted *P* value ≤ 0.05. The bar plot heights represent the coefficients of the linear mixed model, converted to the percentage of difference (percentage of difference= (2ˆβ – 1) × 100) for ease of interpretation (supplemental Table S8).

Within lipid signatures, numerous significant differences in lipid levels between sexes were revealed (for 347 lipid species, adjusted *P* value < 0.05) (Fig. 6, supplemental Table S9). Interestingly, multiple species across several classes showed 5%–30% higher concentrations in females than males. This increase was observed primarily across SPs (n = 69 species), among which 33 SM, 19 Cers, 12 HexCers, and 5 Hex2Cers, and GPs (n = 97 species), represented mainly by ether-linked PC and PE. In contrast, males showed higher concentrations of TGs (n = 140) and lysophospholipids (n = 21).

## Discussion

Quantitative lipid profiling is a powerful phenotyping technique that enables the highly specific analysis (down to fatty acid chain composition, depending on the lipid class) and concentration estimation of a wide panel of circulatory lipid species in more than 20 different lipid classes. These circulatory lipid signatures composed of more than 800 detected, diverse lipid species (contained within lipoproteins for transport) have been recognized as an intermediary phenotype at the molecular level (or endophenotype) which is genetically less complex (or polygenic) than the end-point phenotype (i.e., disease state), but equally heritable (27, 28). In addition to being genetically determined, lipid profiles also integrate the response to internal (e.g., microbiome, inflammation, and oxidative stress) and external (e.g., diet, drug treatment, physical activity, and climate) environmental exposures. As such, when investigated in association with clinical and demographic data, such as age, sex, adiposity markers, blood pressure, markers of diabetes, or even cardiorespiratory fitness, the lipid profiles can facilitate the elucidation of molecular mechanisms behind the revealed associations between the gene variants (i.e., SNPs) and endpoint phenotype(s) or clinical outcome(s) including complex metabolic diseases (e.g., obesity, diabetes, and cardiovascular diseases). Hence, lipidomics has recently gained a considerable interest, specifically in large-scale epidemiological cohort studies.

To minimize the bias introduced through complex sample preparation protocols, and ensure the high throughput, we opted for a single-step extraction with IPA. This method has been evaluated as the best compromise in terms of lipid extraction efficiency, reproducibility and ease of automation, the criteria which were essential for the application in large sample size studies. The application of this method assured the highly reproducible recovery of multiple lipid classes, and through automated sample preparation, we aimed at decreasing the pipetting error, among others, which are associated with manual operators (4, 29, 30). In addition to maximizing the reproducibility of sample preparation, during data acquisition, it was necessary to systematically analyze NIST reference material as external QC sample to correct the signal intensity drift over time. This strategy is widely applied in untargeted assays (31), mainly due to difficulty (or rather impossibility) to cover the entire range of chemically diverse lipids with corresponding ISs. While we performed the IS spike with the widest range possible of commercially available isotopically labeled standards, the external QC and species-specific drift correction strategy has still allowed us to recover 100 additional reproducibly measured lipid species (compared to IS spike alone). This commercially available NIST reference material is not only useful as a long-term QC but it also offers the possibility of reporting the acquired lipid concentrations to its own (consensus) values and thereby harmonize the data and allow for their cross-comparison among different laboratories/studies using different methodologies (32). In our study, we used the NIST plasma QC primarily to further correct for interbatch variability as shown in supplemental Fig. S2. Importantly, plasma materials from different populations with varying ethnic and clinical backgrounds can have different lipid composition and matrix effects. Therefore, the lipid species with higher CVs across external QC material, such as NIST, should not be systematically excluded from further analyses, as their signal within the studied population might be differentally affected by matrix effect. Indeed, multiple PS were robustly detected and quantified in the samples from CoLaus population, whereas they were barely detectable in NIST plasma. The differential matrix effect is also reported on an individual sample basis, as shown by our and some previously reported results (33). While the average lipidome signature, as shown on Fig. 4, is coherent with previous reports, based on relative class and species abundance (7, 34), we observed high individuality of acquired lipid signatures, with significantly lower intraindividual variability in lipid levels compared to interindividual variability. The intra-individual variability of lipid levels is used to determine changes over time within one individual, and inter-individual variability displays how much of the variance observed can be attributed to interparticipant differences. Importantly, for the application of lipidomics in population health research, the between-batch analytical variability must be lower than biological (intra-and inter-individual) variability. Otherwise, the subtle but biologically relevant changes might remain embedded in the analytical noise. Besides, the use of an analyte as a clinical marker is based on the premise that the between-subject variability of this species is greater than the within-individual variability (35). Indeed, as shown in our results, lipid signatures appear to be highly personalized, implying that they serve as “fingerprints” of our chemical individuality, as a reflection of polymorphisms in our DNA sequence and the lifestyle exposures which are unique to each individual (36). This high individuality was also shown in an independent prospective study of 100 individuals (followed for up to 9 years) where the authors highlighted that, in comparison to transcriptome and proteome, but also polar metabolome, the circulatory lipidome is far more personalized (33, 37). Personalized lipid signatures also constitute a prerequisite for patient stratification, in the development of a more precise approach to health monitoring and medical care.

Finally, prior to associating changes in plasma lipidome with specific risk factors or disease, it is crucial to define the baseline variability and “normal” ranges, in apparently healthy populations. It has been reported that sex and age represent the main variables which determine circulatory lipid profiles and their variation over time (9, 38). Therefore, in the first instance, we explored the sex differences across the measured plasma lipidome. Interestingly, more than 50% of the measured lipid species have displayed significant sex differences in their levels (Fig. 6). This suggests important sex specificity and implies sex biases in the metabolism of SPs, ether phospholipids, and glycerolipids (10, 39). Although many among the revealed sex differences are reported here for the first time, multiple findings (specifically at the lipid class level) are consistent with some previously reported differences. For example, elevated concentrations of SMs and PCs were already reported in female individuals in multiple studies (10, 33, 40). While the origin of these differences remains to be further investigated, we hypothesize that they are likely sex hormone driven and a result of differential activity of specific enzymes involved in the lipid metabolism. The sex-specific enzymatic activities were reported for LCAT and phospholipase A2, for example, and could explain the observed sex differences related to phospholipid classes (41, 42). The significant sex-dependent changes with aging were already reported for these lipid classes including the ether-linked species (10, 40). However, it is important to note that a significant proportion of previous reports systematically considered the patients with acquired metabolic diseases. Consequently, the sex differences revealed in these previous studies might have been biased by the disease-altered lipid profiles.

Importantly, in the investigated population most females were already in postmenopause (n = 130) at the baseline. In total, only nine women were using contraceptives, while 51 females in postmenopause were undergoing hormonal replacement therapy. While the hormonal therapy and contraceptives have been proven to have a significant effect on increased lipid levels in female blood plasma (43), in the present study, the observed sex differences were confirmed even after the exclusion of females taking various hormonal supplements (data not shown).

Overall, the described sex differences at the lipid species level and the degree of sex-bias within the circulatory lipidome call for the necessity of further investigation and consideration of sex differences for cardiometabolic risk assessment, clinical diagnostics, and treatment.

## Limitations

The presented study focused on the evaluation of batch-to-batch measurement precision, as well as individuality (within versus between-subject variability) and sex specificity of acquired circulatory lipid profiles. Thus, serving as a basis for their valorization for population stratification and, thereby, clinical utility for improved risk prediction and diagnostics. More advanced and detailed statistical exploration, including association and causality analysis with clinical outcomes (anthropometry, diabetes and adiposity markers, physiological parameters, etc.) will be done on the entire dataset when acquired (>7,000 samples) for maximal statistical power.

Regarding lipid data, it should be noted that the concentrations of neutral lipids, specifically TGs and CEs, were estimated based on IS containing saturated fatty acyls. Considering the ionization and fragmentation efficiency of these neutral lipid species is strongly dependent on their fatty acid composition, the concentration estimation can be further improved by the calculation of response factors.

## Data availability

The data relevant for the developed measurement workflow (targeted lipid species with corresponding transitions and ISs), analytical variability, mean concentrations of measured lipids, biological variability, and sex differences are contained within the article. The clinical (demographic) data of CoLaus|PsyCoLaus study used in this article cannot be fully shared as they contain potentially sensitive personal information on participants. According to the Ethics Committee for Research of the Canton of Vaud, sharing these data would be a violation of the Swiss legislation with respect to privacy protection. However, coded individual-level data (including lipid concentrations) that do not allow researchers to identify participants are available upon request to researchers who meet the criteria for data sharing of the CoLaus|PsyCoLaus Datacenter (CHUV, Lausanne, Switzerland). Any researcher affiliated to a public or private research institution who complies with the CoLaus|PsyCoLaus standards can submit a research application to research.colaus@chuv.ch or research.psycolaus@chuv.ch. Proposals will be evaluated by the Scientific Committee of the CoLaus|PsyCoLaus study. Detailed instructions for gaining access to the CoLaus|PsyCoLaus data used in this study are available at www.colaus-psycolaus.ch/professionals/how-to-collaborate/.

## Supplemental data

This article contains supplemental data.

## Conflict of interest

The authors declare that they have no conflicts of interest with the contents of this article.
