# Supplementary material for: Clinical lipidomics reveals high individuality and sex specificity of circulatory lipid signatures: a prospective healthy population study
Source: J Lipid Res. 2025 Mar 18;66(5):100780. doi: 10.1016/j.jlr.2025.100780 (PMC12022646; doi:10.1016/j.jlr.2025.100780)
Supplement: Supplementary Information [file mmc2.docx]

**Supplementary Information for**

**Clinical Lipidomics Reveals High Individuality and Sex Specificity of Circulatory Lipid Signatures: A Prospective Healthy Population Study**

Jessica Medina^1#^, Nicolas Goss^1#^, Gonçalo dos Santos Correia^2,3^, Rebecca Borreggine^1^, Tony Teav^1^, Zoltan Kutalik^5^, Pedro Marques Vidal^4^, Hector Gallart-Ayala^1^*, Julijana Ivanisevic^1^*

^1^Metabolomics Platform, Faculty of Biology and Medicine, University of Lausanne, Quartier UNIL-CHUV, Rue du Bugnon 19, CH-1005 Lausanne, Switzerland

^2^Institute of Reproductive and Developmental Biology, Department of Metabolism, Digestion and Reproduction, Imperial College London, London W12 0NN, UK

^3^March of Dimes Prematurity Research Centre at Imperial College London, London, UK

^4^Department of Medicine, Internal Medicine, Lausanne University Hospital (CHUV) and University of Lausanne, Lausanne, Switzerland

^5^Department of Computational Biology, Faculty of Biology and Medicine, University of Lausanne, Switzerland

^#^Equal contribution

*Corresponding authorship (for correspondence: Julijana.ivanisevic@unil.ch, +41216925098)

**SHORT TITLE**

**Individuality and Sex Specificity of Circulatory Lipidome**

# Experimental section

Chemicals and reagents.

LC-MS grade water and organic solvents (acetonitrile and 2-propanol (IPA)) and ammonium acetate were purchased from Biosolve Chimie (Dieuze, France) and Sigma-Aldrich (Darmstadt, Germany), respectively. UltimateSplash™ One mixture comprising sixty-nine internal standards and individual internal standards Glucosyl(β) Ceramide (d18:1/15:0)-d7, Lactosyl(β) Ceramide (d18:1/15:0)-d7 and dihydroceramide (d18:1/13:0)-d7 were purchased from Avanti Polar Lipids (Alabaster, AL, USA). In addition, fatty acid standards including arachidonic acid (20:4)-d8, docosahexanoic acid (22:6)-d5 and eicosapentaenoic acid (20:5)-d5 were purchased from Sigma Aldrich (Darmstadt, Germany).

Internal standard mixture preparation.

The internal standard mixture was prepared by mixing 840 µL of UltimateSplash™ One (provided in Dichloromethane: Methanol (DCM:MeOH 1:1)), 700 µL of deuterated ceramide (Glucosyl(β)-Ceramide (d18:1/15:0)-d7, Lactosyl(β)-Ceramide (d18:1/15:0)-d7, dihydroceramide (d18:1/13:0)-d7) and free fatty acid solution in DCM:MeOH (1:1) (linoleic acid (18:2)-d4, arachidonic acid (20:4)-d8, docosahexanoic acid (22:6)-d5, eicosapentaenoic acid (20:5)-d5). This final mixture was diluted (1/20)(*v/v*) with 2-propanol. Concentrations of each lipid in the IS mixture are reported in **Table S1**.


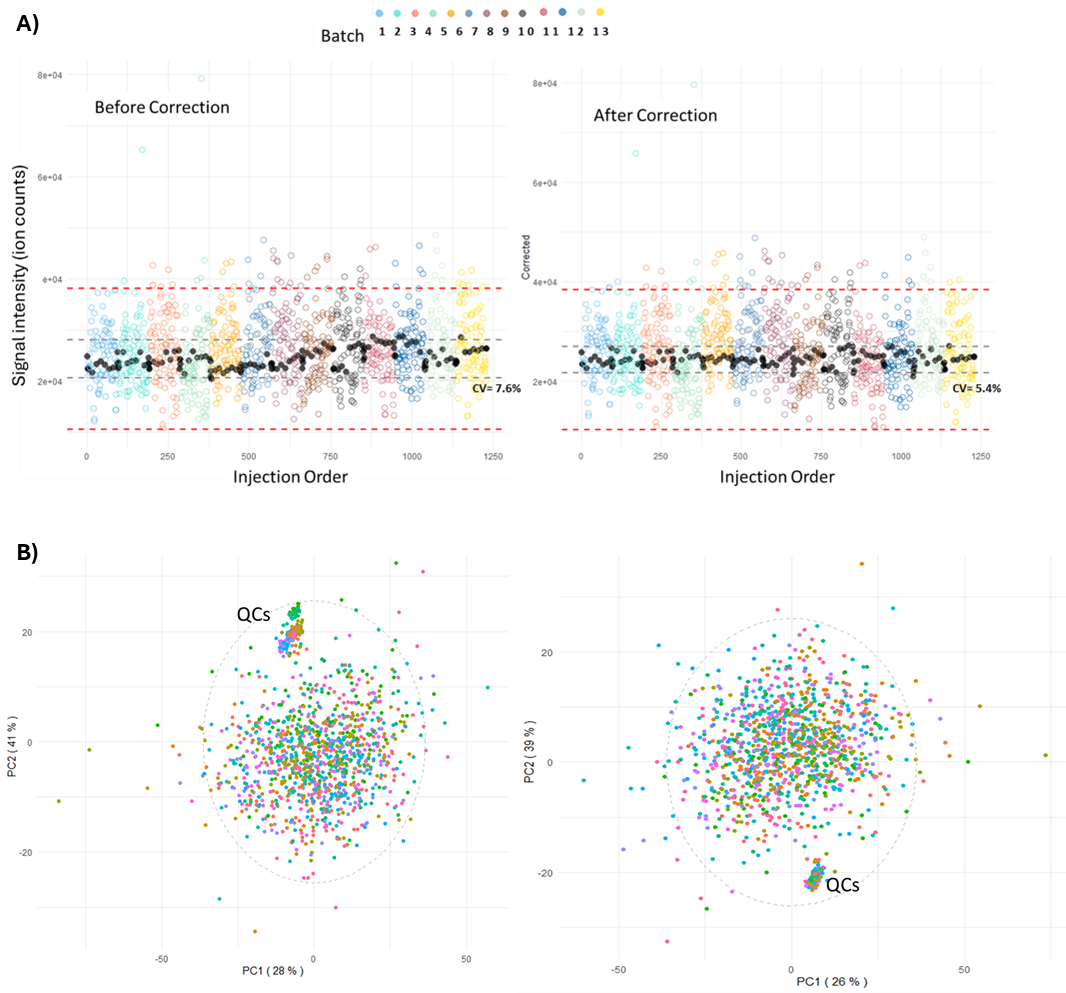


**Figure S1. Between-batch signal intensity drift correction**. **A)** Time series plots showing signal intensity of PC 18:0_20:4 across all QCs (deep colored circles) and over 13 batches. Left, before signal intensity drift correction. Right, after signal intensity correction. **B)** Score plots as outcome of PCA, before and after signal intensity drift correction. See QC grouping.

**
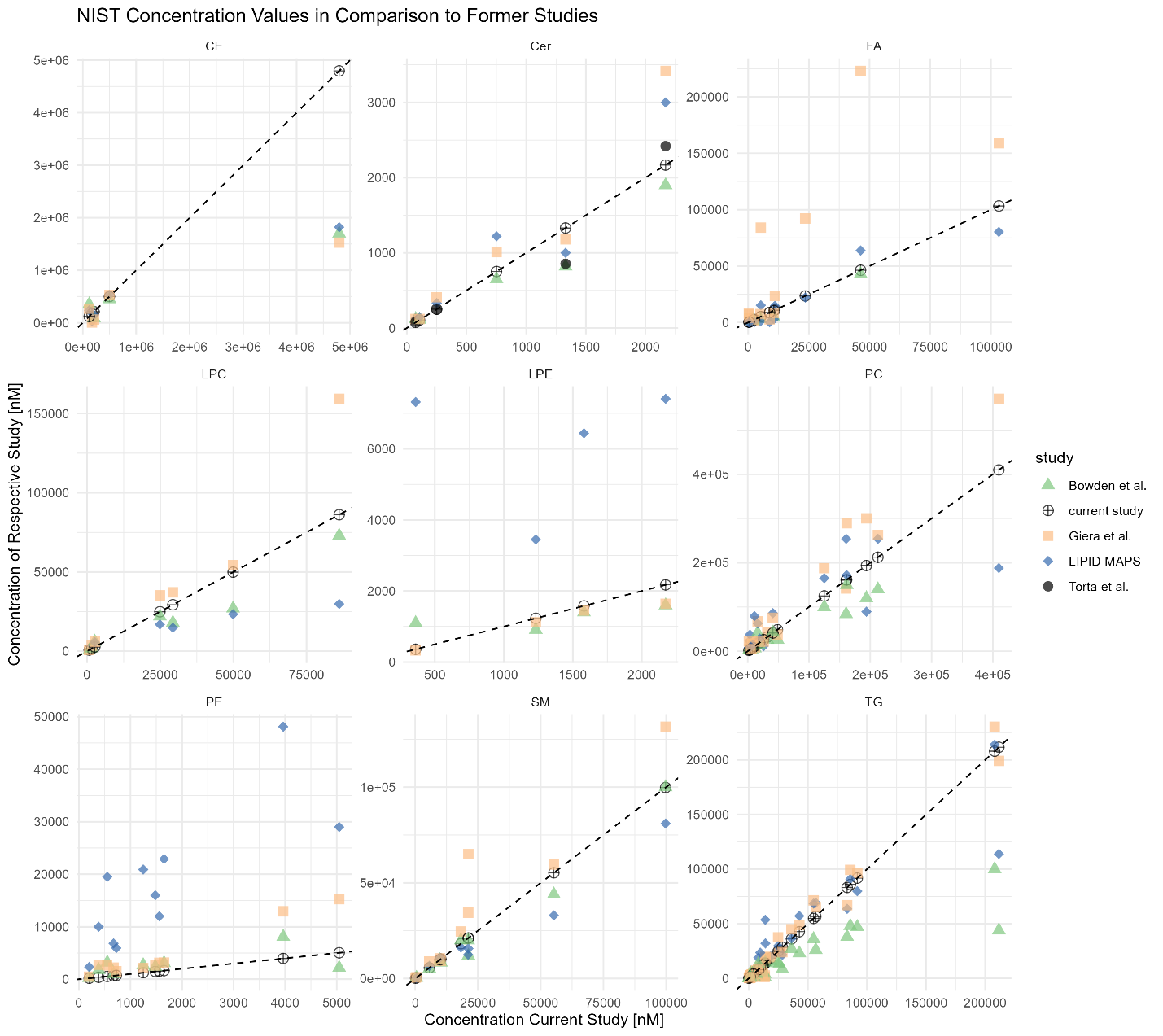
Figure S2.** Comparison of reported concentration of lipid species in NIST SRM 1950 plasma between several former inter-laboratory studies (Ghorasaini et al. Lipidyzer study 2021, LIPID MAPS 2010 study, Bowden et al. 2017, Torta et al. 2024) and current study.

**Figure S3.** Plasma lipidome variability based on intra-class correlation coefficients (ICCs) for lipid subclasses measured over three follow-ups.
